# Effects of Body Mass and Temperature on Sexual Selection in Bumblebees (*Bombus terrestris*) Under Equal Sex Ratios

**DOI:** 10.3390/insects17050481

**Published:** 2026-05-08

**Authors:** Min Su Park, Ji Hyun Woo, Weiyue Qiu, Hyung Joo Yoon, Bo Yeon Kim, Kyeong Yong Lee, Kwang Sik Lee, Byung Rae Jin

**Affiliations:** 1 College of Natural Resources and Life Science, Dong-A University, Busan 49315, Republic of Korea; parkminsu100@naver.com (M.S.P.); jihyun3282@naver.com (J.H.W.); qiuweiyue1112@163.com (W.Q.); yoonhj1023@dau.ac.kr (H.J.Y.); boyeon@dau.ac.kr (B.Y.K.); 2 Department of Agricultural Biology, National Institute of Agricultural Sciences, Wanju 55365, Republic of Korea; ultrataro@korea.kr

**Keywords:** body size, bumblebee, climate change, insect pollinator, mating, sexual selection

## Abstract

Bumblebees are key pollinators in agricultural and natural ecosystems; however, climate change is driving shifts in population size, body size, and diversity. Accelerating global warming affects bumblebee body size, thereby influencing the strength of sexual selection and overall population fitness. Thus, this study aimed to investigate the effects of body mass and temperature on sexual selection in *Bombus terrestris* reared under warm conditions, with mating conducted between equal sex ratios at both optimal (23 °C) and elevated (32 °C) temperatures. Mating success was lower at 32 °C than at 23 °C, regardless of body mass; however, larger queens and males consistently exhibited higher mating success at both temperatures than their smaller counterparts. Mate-choice patterns were similar across temperatures: large queens predominantly mated large males, whereas small ones mainly mated small males. However, small queens tended to mate large males at 32 °C than at 23 °C. Meanwhile, matings between small queens and small males occurred relatively later at both temperatures. Although small males exhibited longer mating durations than large ones, the amount of sperm transferred to the spermathecae of the queens was positively associated with the body size of both males and queens. Our findings indicate that, at equal sex ratios, mate choice in bumblebees is primarily determined by body mass at all temperatures. Thus, smaller bumblebees associated with higher temperatures had the lowest mating success.

## Simple Summary

Understanding how temperature and body mass affect mating behavior, sexual selection, and selection outcomes in bumblebees is critical for predicting population fitness under future climate warming. Therefore, we investigated patterns of sexual selection on body mass in bumblebees under equal sex ratios at both optimal and elevated temperatures. We provide the first evidence that under equal sex ratios across temperatures, sexual selection in bumblebees depends on the body mass of both queens and males. We found that higher temperatures and smaller body size reduced mating rates in bumblebees, indicating that body mass and temperature strongly influence sexual selection and mating success. Our findings highlight the strong effects of body mass and temperature on selection and mating success in bumblebees and represent an important aspect to consider in the context of future climate warming.

## 1. Introduction

Climate warming is accelerating globally at local and regional scales, thereby threatening biodiversity, population stability, and increasing the risk of species extinction [1]. In particular, frequent and prolonged heatwaves represent a severe threat to the populations and diversity of cold-adapted bumblebees [2]. Notably, bumblebees, which are crucial pollinators in agricultural and natural ecosystems in temperate regions, are experiencing climate-induced changes in population size, body size, and diversity [3]. Although bumblebees have shown plastic and adaptive responses to climate change in various traits [3], elevated temperatures resulting from climate warming have been shown to have negative impacts on the morphology and behavior of bumblebee populations [4,5,6,7,8,9] and have contributed to declines in biodiversity and populations [10,11,12,13,14].

Elevated developmental temperatures have been shown to reduce body size in bumblebees [3,8,9,15,16], which lowers mating rates and decreases hibernation survival [17], ultimately resulting in population declines. In addition, bumblebees under elevated temperatures exhibited a trade-off between increasing colony size and reducing body size [9,18], as well as changes in the mating behaviors between queens and males [17]. In bumblebees, strong mate choice for large males and queens under competitive conditions at elevated temperatures result in the transfer of sufficient sperm from large males and high post-hibernation survival of large queens [17]. This could imply that a preference for larger body size at elevated temperatures is positively correlated with fitness proxies [19], such as fecundity and survival [20,21,22]. Therefore, changes in the mating preferences of queens and males under elevated mating temperatures may reflect the positive role of sexual selection in promoting evolutionary adaptation in bumblebees [23].

Sexual selection promotes the evolution of adaptive traits [22,23,24]. A meta-analysis indicated that sexual selection positively affects population fitness under changing environmental conditions [23]. Additionally, precopulatory sexual selection, including mate competition and mate choice, promotes thermal adaptation and evolutionary rescue [25]. Understanding how mating temperatures affect mating behaviors, sexual selection, and the reproductive success in bumblebees is critical [9,17]. Indeed, given the negative relationship between elevated developmental temperatures and morphological traits in bumblebees [8], as well as trade-offs between colony size and body size [9,18], an important question is whether the increased variation in bumblebee body size influences sexual selection under elevated mating temperatures.

The optimal developmental temperature for bumblebees (*Bombus terrestris*) is approximately 27–29 °C [26,27,28,29], and their optimal mating temperature is approximately 23 °C [9,17]. Under these conditions, queens of *B. terrestris* mate males [30] and then overwinter underground. Meanwhile, temperatures at or above 32 °C cause thermal stress in bumblebees [4,5,6,7,15,16] and are considered indicative of heatwaves [14,29], resulting in reduced body size and decreased pollination activity [5,6,8,16,31,32,33,34]. Mating in bumblebees is a critical event in the life cycle and is influenced by factors such as sex ratio, age, temperature, and body size [9,17,35,36,37]. Body size and ambient temperature during the mating period are important determinants of mating success [38,39,40,41,42]. A preference for larger mates is a widespread reproductive strategy in many animal species, including insects [43,44,45,46].

Given the globally accelerating warming, understanding how climate change affects mating and sexual selection is crucial for predicting the responses of species to future climate change [47,48]. Our previous studies on bumblebees (*B. terrestris*) have shown that both body size and mating rates decrease at elevated temperatures [8,9,17]. Experimental results on mate-choice preferences in bumblebees reared under warm conditions further revealed that, under competitive conditions with different sex ratios, large males have higher mating success than small ones at both optimal and elevated temperatures. In contrast, male preference for large queens is observed predominantly at elevated temperatures rather than at optimal temperatures [17]. This study aimed to investigate mate-choice preferences on body mass in bumblebees under mating conditions with equal sex ratios by presenting equal numbers of newly emerged queens and males at both optimal and elevated temperatures. Our findings demonstrate an interaction between body mass and temperature in shaping mating behavior, mating success, and reproductive outcomes in bumblebees reared under warm conditions. These experimental results may serve as a model for predicting bumblebee population fitness under future climate warming.

## 2. Materials and Methods

### 2.1. Bumblebee Rearing

*Bombus terrestris* colonies were successively reared in an indoor system [28] at the Department of Applied Biology at Dong-A University. Bumblebees, designated for warming experiments as previously described [17], underwent four to five generations of indoor rearing at 32 °C and were maintained in a room at 32 °C and 65% relative humidity under continuous darkness [9]. Bumblebees were fed with a mixture of pollen and a 40% sucrose solution ad libitum. To provide sufficient mate-choice opportunities for queens, which typically mate with only a single male [30], mating trials were conducted using more than 10 newly emerged queens (6–8 days after emergence) and 20–30 newly emerged males (6–8 days after emergence). All individuals were collected from different colonies to prevent inbreeding [9,37]. Mating and hibernation procedures for bumblebees were performed as described in our previous studies [9,49]. Mating between newly emerged queens and males was conducted in wooden mating boxes with steel-mesh sides. A large mating box (60 × 50 × 65 cm) was used for approximately 100 individuals, whereas a small mating box (46 × 46 × 47 cm) was used for no more than 60 individuals. All mating trials were conducted at 23 °C for 1 week under 1000 lux of lighting and a 14 L:10 D photoperiod, with pollen bread and a 40% sucrose solution provided [9]. Mated queens were then separated and placed in ventilated plastic boxes at 10–15 °C for one day. To induce hibernation conditions, the mated queens were weighed, transferred to ventilated tubes, and stored in incubators at 2.5 °C and 70% relative humidity under constant darkness for 12 weeks [49]. After hibernation, the queens were placed in a flight box (60 × 50 × 65 cm) at 23–25 °C for 3 days to induce flight orientation [9]. The queens that had undergone hibernation were individually transferred to ventilated wooden nesting boxes (9.5 × 15.0 × 10.5 cm) to initiate oviposition. The nesting boxes for colony development were replaced according to the number of newly emerged workers: after 5–10 workers had emerged, the oviposition plates were transferred to ventilated plastic nesting boxes (15.5 × 16.5 × 10.5 cm), and after 40–50 workers had emerged, the nests were moved to larger boxes (22.0 × 28.0 × 14.0 cm). Newly emerged queens and males were collected from over 50 colonies and used in subsequent experiments.

### 2.2. Mating Experiment

**Figure 1:**
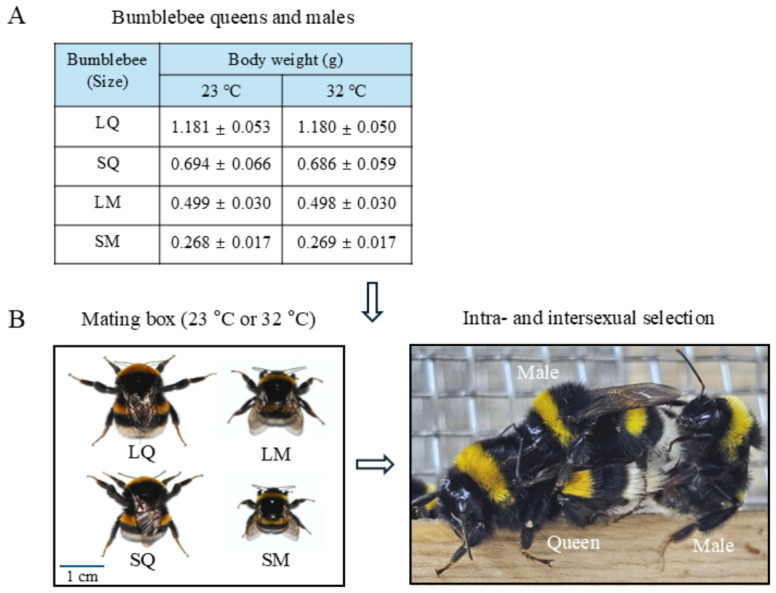
Mating experiments. (**A**) Groups of males and queens by body mass. Large and small queens (LQs and SQs, respectively) and males (LMs and SMs, respectively) were classified based on body weight measured 6–8 days after emergence at a constant temperature of 32 °C. The average body masses of the bumblebees used in both the 23 °C and 32 °C experiments are shown. (**B**) For intra- and intersexual selection experiments, the treatment groups consisted of LQs, SQs, LMs, and SMs at either 23 °C (optimal mating temperature) or 32 °C (elevated mating temperature). Mating experiments were conducted in a mating box with equal sex ratios and equal numbers of LQs, SQs, LMs, and SMs. Representative images of males and queens during mating competition are provided. The total sample size for each group was *n* = 106.

For the mating success experiments, newly emerged queens (6–8 days post-emergence) and males (6–8 days post-emergence) were collected from more than 50 colonies that were reared indoors at 32 °C. The weights of the newly emerged queens and males were individually measured by securing each queen and male in a transparent tube cap without anesthesia and using an electronic scale (AE260 Delta Range, Mettler Toledo, Columbus, OH, USA). The newly emerged queens and males reared indoors at 32 °C were divided into four groups based on body weight (Figure 1A): large (LQ) and small (SQ) queen groups, and large (LM) and small (SM) male groups. For the mating success analyses in the mating experiments with equal sex ratios (Figure 1B), we assessed mate- choice preferences by introducing equal numbers of individuals from the LQ, SQ, LM, and SM groups (*n* = 11–13 per group; total = 106 per group; nine replicates per treatment). In each replicate, equal numbers of individuals from the LQ, SQ, LM, and SM groups were placed in a wooden mating box (46 × 46 × 47 cm) with steel-mesh sides. Mating experiments were conducted at optimal (23 °C) and elevated (32 °C) temperatures in humidity-controlled incubators under 1000 lux illumination. During the mating experiments, the mating boxes contained a mixture of pollen bread and a 40% sucrose solution. After mating initiation, copulating queens and males were transferred to ventilated wooden nesting boxes (9.5 × 15.0 × 10.5 cm) in separate incubators under the same conditions. The mating occurrence time and mating duration were recorded. After mating was completed, queens and males were weighed again to assess body mass. Mating experiments were observed for 120 min, considering the decreased opportunity for selection resulting from the reduction in population variance associated with mating success [50]. The mating rates between LQ or SQ and LM or SM were presented as the proportion of mated individuals relative to the total individuals in each replicate. Additionally, mating experiments were performed by introducing equal numbers of LQ and LM (total = 379 per group, 11 replicates) or SQ and SM (total = 105 per group, 7 replicates) into wooden mating boxes appropriately sized for the population at 32 °C. Mating rates were recorded as described above.

### 2.3. Sperm Counting

To assess the relationship between mating behavior and sperm transfer, sperm counting procedures were performed as described in our previous study [17]. After the completion of mating at 23 °C and 32 °C, the spermathecae and seminal vesicles of queens and males were dissected using a stereomicroscope (Zeiss, Jena, Germany). Seminal vesicles from mated males were dissected individually 6 h after mating, and seminal fluid containing sperm was extracted by gently pressing the dissected vesicles. Spermathecae were dissected individually from mated queens 24 h after mating, and spermathecal fluid containing sperm was extracted by pressing the spermathecae. For the sperm counting evaluation, the seminal and spermathecal fluids were diluted with phosphate-buffered saline (pH 7.4), and sperm numbers were counted using a hemocytometer under a light microscope (Optinity 4K HD Camera KCX-80LA, Olympus, Tokyo, Japan). Total sperm quantities were estimated based on the number of sperm remaining in the seminal vesicles after mating and the number of sperm transferred to the spermathecae following mating.

### 2.4. Statistical Analysis

The data were statistically analyzed using SPSS version 22.0 (IBM Inc., Chicago, IL, USA) and are presented as the mean ± standard deviation (SD). A one-way analysis of variance (ANOVA) followed by Tukey’s honestly significant difference (HSD) test was used to determine statistical significance, and a Shapiro–Wilk test was applied to assess data normality. Comparisons of cumulative mating rates between 23 °C and 32 °C were performed using the Wilcoxon rank-sum test for non-normally distributed data and a two-sample *t*-test for normally distributed data. The *p*-values are reported as *p* < 0.05 (*), *p* < 0.01 (**), and *p* < 0.001 (***).

## 3. Results

### 3.1. Mating Experiment

**Figure 2:**
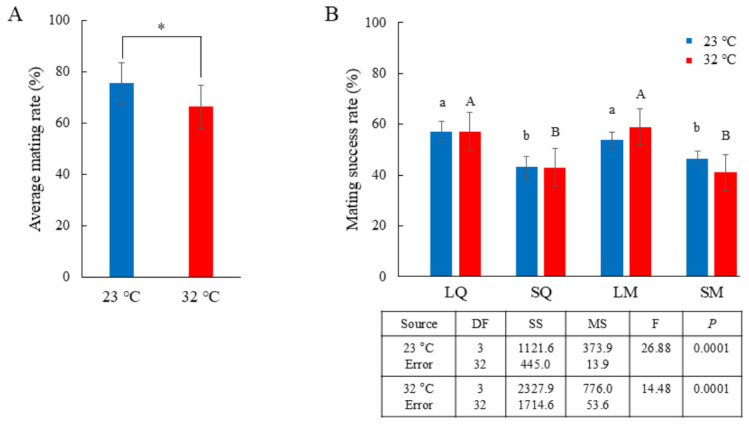
Mating rates under equal sex ratios with equal numbers of LQs, SQs, LMs, and SMs. (**A**) Average mating rates (%) at 23 °C and 32 °C (*n* = 11–13 per group; total *n* = 106 per group; nine replicates per treatment). Statistical significance was determined in a *t*-test; *, *p* < 0.05. (**B**) Mating success rates (%) of LQs, SQs, LMs, and SMs at 23 °C and 32 °C, partitioned by group and normalized to 100% of the average mating rate, are shown in (**A**). Different letters indicate significant differences among treatments (one-way ANOVA, *p* = 0.0001 at both 23 °C and 32 °C).

In the equal sex ratio mating experiments, conducted using equal numbers of individuals from the LQ, SQ, LM, and SM groups, the mating experiment was assessed at optimal and elevated temperatures of 23 °C and 32 °C (Figure 1). The average mating rates were lower at 32 °C than at 23 °C: approximately 66% of individuals mated at 32 °C, compared with approximately 75% at 23 °C (*t* = 2.360, df = 15; *p* < 0.05; Figure 2A). When mated individuals were categorized by body mass, the LQ and LM groups exhibited significantly higher mating success than the SQ and SM groups at both temperatures (23 °C: F_3,32_ = 8.25, *p* = 0.0001; 32 °C: F_3,32_ = 8.44, *p* = 0.0001; Figure S1). However, the rate of mating success for all groups (LQ, SQ, LM, and SM) was lower at 32 °C than at 23 °C (Figure S1). When average mating rates were normalized to 100% within the LQ–SQ and LM–SM groups and partitioned into the LQ, SQ, LM, and SM groups (Figure 2A), mating success in LQ and SQ showed similar patterns, with approximately 57% in LQ and approximately 43% in SQ at both temperatures. In contrast, LM exhibited higher mating success at 32 °C than at 23 °C, whereas SM showed lower mating success at 32 °C (23 °C: F_3,32_ = 26.88, *p* = 0.0001; 32 °C: F_3,32_ = 14.48, *p* = 0.0001; Figure 2B).

**Figure 3:**
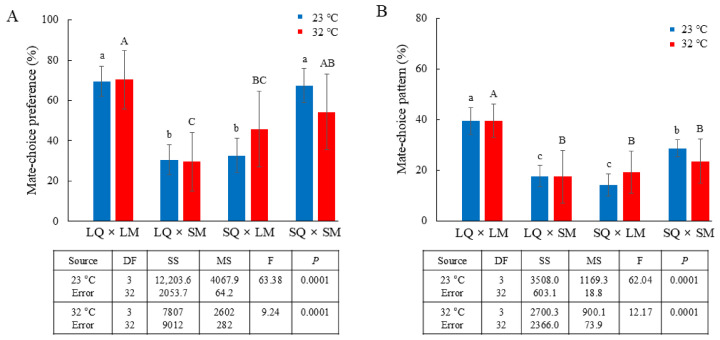
Mate choice under equal sex ratios with equal numbers of LQs, SQs, LMs, and SMs. (**A**) Mate-choice preference (%) of LQs with LMs or SMs and SQs with LMs or SMs at 23 °C and 32 °C, partitioned by LQ and SQ mating preferences and normalized to 100% of the cumulative mating rates, shown in Figure 2A. Different letters indicate significant differences among treatments (one-way ANOVA, *p* = 0.0001 at both 23 °C and 32 °C). (**B**) Mate-choice pattern (%) of LQs with LMs or SMs and SQs with LMs or SMs at 23 °C and 32 °C, normalized to 100% of the mate-choice patterns of the total groups shown in (**A**). Different letters indicate significant differences among treatments (one-way ANOVA, *p* = 0.0001 at both 23 °C and 32 °C).

Mate-choice preferences based on the LQ and SQ mating success rates were consistent at both temperatures: LQ–LM matings occurred at significantly higher rates, followed by SQ–SM matings (23 °C: F_3,32_ = 63.38, *p* = 0.0001; 32 °C: F_3,32_ = 9.24, *p* = 0.0001; Figure 3A). At both temperatures, approximately 70% of LQs mated with LMs, while 30% mated with SMs. At 23 °C, approximately 33% of SQs mated with LMs and 67% mated with SMs, whereas at 32 °C, SQs exhibited a different mate-choice preference, with approximately 46% mating with LMs and 54% with SMs. Interestingly, SQ–SM matings were less frequent at 32 °C than at 23 °C, and SQ individuals exhibited a stronger tendency to mate with LMs at 32 °C than at 23 °C. This suggests that SMs represent a disadvantageous mating partner, especially at higher temperatures.

When the mate-choice patterns of LQs with LMs or SMs and those of SQs with LMs or SMs were normalized to a total of 100%, the frequencies of LQ–LM matings (approximately 39%) and LQ–SM matings (approximately 18%) remained consistent across both temperature conditions. In contrast, the selection patterns of SQs at 32 °C differed from those at 23 °C: SQ–LM matings were approximately 14% at 23 °C and 19% at 32 °C, whereas SQ–SM matings were approximately 29% at 23 °C and 24% at 32 °C (23 °C: F_3,32_ = 62.04, *p* = 0.0001; 32 °C: F_3,32_ = 12.17, *p* = 0.0001; Figure 3B). Overall, the LQ–LM mating pairings showed a predominant selection pattern at both temperatures, although mating rates decreased at the higher temperature.

Specifically, the frequency of SQ–SM matings at 32 °C was lower than expected based on selection patterns. To further examine this observation, we conducted independent mating experiments using equal numbers of SQs and SMs or LQs and LMs at 32 °C, and found that SQ–SM matings (approximately 34%) were significantly lower than LQ–LM matings (approximately 86%) (*t* = 38.61, df = 15; *p* = 0.0001; Figure S2). This indicates that SQs and SMs exhibit less mate-choice preference at higher temperatures.

### 3.2. Selection Outcomes

**Figure 4:**
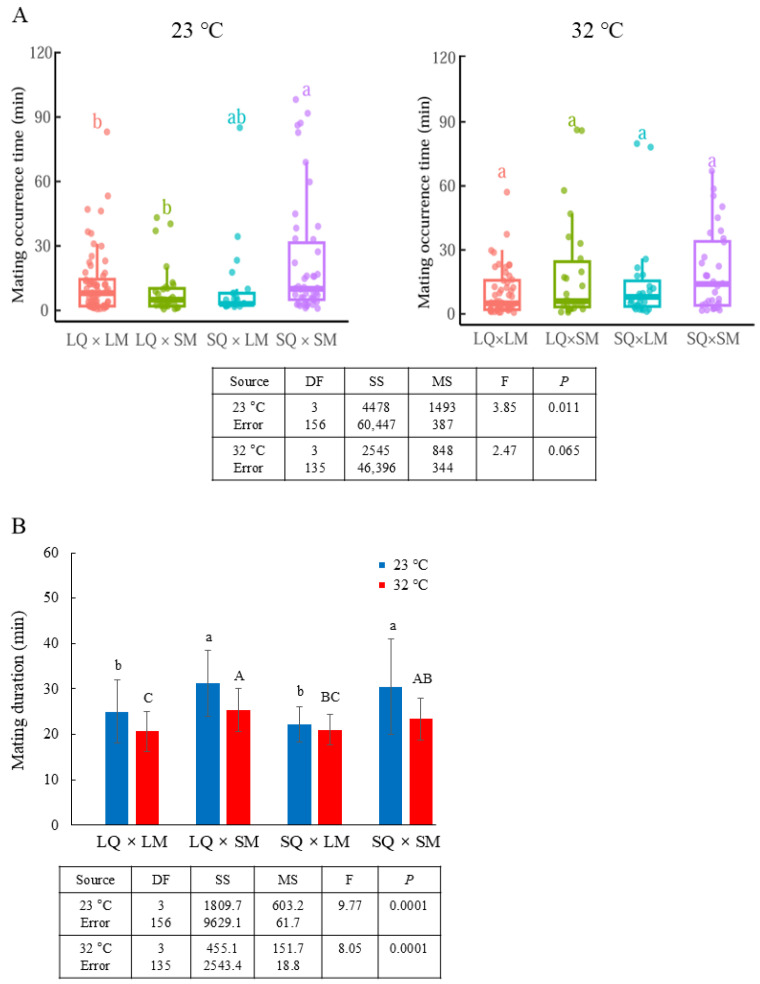
Mating behavior in mate-choice patterns. (**A**) Timing of mating occurrences of LQs with LMs or SMs and SQs with LMs or SMs at 23 °C (total *n* = 63 for LQ–LM, 28 for LQ–SM, 23 for SQ–LM, and 46 for SQ–SM) and at 32 °C (total *n* = 53 for LQ–LM, 26 for LQ–SM, 27 for SQ–LM, and 33 for SQ–SM). Mating occurrences were recorded over 2 h. Different letters indicate significant differences among treatments (one-way ANOVA, *p* = 0.011 at 23 °C and *p* = 0.065 at 32 °C). (**B**) Mating durations of LQs with LMs or SMs and SQs with LMs or SMs at 23 °C (total *n* = 63 for LQ–LM, 28 for LQ–SM, 23 for SQ–LM, and 46 for SQ–SM) and at 32 °C (total *n* = 53 for LQ–LM, 26 for LQ–SM, 27 for SQ–LM, and 33 for SQ–SM). Different letters indicate significant differences among treatments (one-way ANOVA, *p* = 0.0001 at both 23 °C and 32 °C).

In the selection patterns, SQ–SM matings occurred later at both temperatures (23 °C: F_3,156_ = 3.85, *p* = 0.011; 32 °C: F_3,135_ = 2.47, *p* = 0.065; Figure 4A). At 32 °C, the average mating occurrence time of LQ–LM matings was faster than that of the other groups and also faster than at 23 °C; however, the difference was not statistically significant. At both temperatures, SMs exhibited significantly longer mating durations than LMs, regardless of whether the mating partner was a LQ or SQ (23 °C: F_3,156_ = 9.77, *p* = 0.0001; 32 °C: F_3,135_ = 8.05, *p* = 0.0001; Figure 4B and Table S1). Overall, mating durations at 23 °C tended to be longer than those at 32 °C.

**Figure 5:**
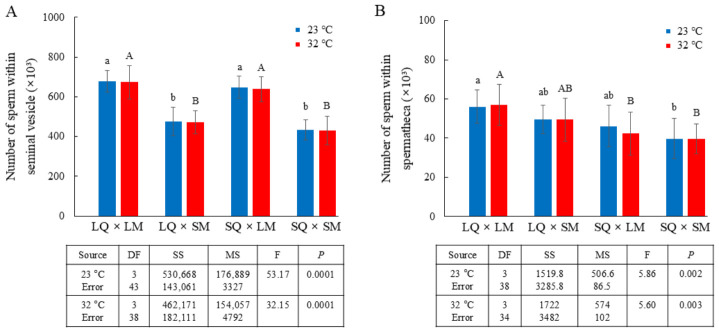
Transfer of sperm from males to queens and storage in the spermathecae of queens through mating. (**A**) Number of sperm remaining in the seminal vesicles of LMs after mating with a LQ or SQ and SMs after mating with a LQ or SQ at 23 °C (total *n* = 14 for LQ–LM, 10 for LQ–SM, 12 for SQ–LM, and 11 for SQ–SM) and at 32 °C (total *n* = 11 for LQ–LM, 11 for LQ–SM, 9 for SQ–LM, and 11 for SQ–SM). Different letters indicate significant differences among treatments (one-way ANOVA, *p* = 0.0001 at both 23 °C and 32 °C). (**B**) Number of sperm in the spermathecae of LQs after mating with a LM or SM and SQs after mating with a LM or SM at 23 °C (total *n* = 11 for LQ–LM, 10 for LQ–SM, 10 for SQ–LM, and 11 for SQ–SM) and at 32 °C (total *n* = 10 for LQ–LM, 10 for LQ–SM, 9 for SQ–LM, and 9 for SQ–SM). Different letters indicate significant differences among treatments (one-way ANOVA, *p* = 0.001 at both 23 °C and 32 °C).

To compare selection outcomes across selection patterns, we counted the number of sperm in the seminal vesicles of males and the spermathecae of queens after mating (Figure 5 and Table S1). Across both temperatures, the LMs possessed a higher remaining sperm count in the seminal vesicles after mating (averaging approximately 638,095–678,359) than the SMs (averaging approximately 430,195–475,000) (23 °C: F_3,43_ = 53.17, *p* = 0.0001; 32 °C: F_3,38_ = 32.15, *p* = 0.0001; Figure 5A and Table S1). Consistent with mating duration at both temperatures, the LMs transferred approximately 7.63–7.80% of their sperm to the LQs and 6.21–6.64% to the SQs, whereas the SMs transferred approximately 9.44–9.48% to the LQs and 8.38–8.46% to the SQs (Table S1). However, the LQs stored approximately 56,039–57,000 sperm from the LMs and 49,429–49,571 from the SMs, while the SQs stored approximately 42,302–46,143 sperm from the LMs and 39,740–39,762 from the SMs (Figure 5B and Table S1). Collectively, the amount of sperm transferred to the spermathecae of the queens was positively associated with male body size—larger males transferred more sperm—and with queen body size—larger queens stored more sperm (23 °C: F_3,38_ = 5.86, *p* = 0.002; 32 °C: F_3,34_ = 5.60, *p* = 0.003; Figure 5B).

## 4. Discussion

Globally accelerating climate change is threatening the diversity and population of bumblebees [2,3]. Thus, to assess thermal adaptation to climate warming, we continuously reared bumblebee colonies under warm conditions [8,9,17]. Based on observations of developmental characteristics and mating behaviors at various temperatures, we determined that a temperature of 32 °C represents the upper thermal limit for bumblebee colony development [8,9,17]. This study examined how body mass and temperature can influence in bumblebees by conducting mating experiments with equal sex ratios and investigating the resulting patterns at optimal and elevated temperatures. Considering that colony size in bumblebees increases with greater variation in body size at elevated temperatures [8,9,18], mating behavior between queens and males across a range of body masses under different temperature conditions is an important consideration in the context of future climate warming. We show that mating success was lower when temperatures were elevated from an optimal temperature of 23 °C to 32 °C, regardless of whether bumblebee colonies were reared at 32 °C or mating occurred under equal or biased sex ratios [17]. Notably, the mating success of individuals at 32 °C was lower than that at 23 °C, regardless of body mass. This significant decrease in mating rates at higher temperatures indicates that mating success in bumblebees is strongly influenced by temperature [9,17]. At higher temperatures, pre-copulatory mating behaviors such as male–male competition for larger queens [17] and mating avoidance by queens [9] have been reported. Indeed, temperature influences various traits in bumblebees, including mating behavior [9,17], foraging behavior [5], cognitive performance [4], sensory processing [7], and body size [8,18]. Temperature also affects male mating signals and female choice in mason bees [51]. Thus, elevated temperatures may strongly affect mating success in bumblebees and alter subsequent behavioral responses.

In mating experiments involving bumblebees of different body masses (LQs, SQs, LMs, and SMs) under equal sex ratios, larger queens and males exhibited significantly higher mating success than their smaller counterparts at both optimal and elevated temperatures. These results indicate that the body mass of bumblebee queens and males strongly affects mating success. Bumblebee mate-choice experiments revealed that large males achieve higher mating success than small males at both optimal and elevated temperatures, whereas a strong male preference for large queens is exhibited at elevated temperatures [17]. This also suggests assortative mating by size, a common pattern in natural populations [52]. Overall, these results indicate that under equal sex ratios, larger queens and males exhibit greater mating success at both optimal and elevated temperatures.

Under equal sex ratio conditions, large queens showed stronger mate-choice preferences for large males, whereas small queens frequently mated with small males. Consequently, matings between large queens and large males exhibited the strongest selection patterns at both temperatures, followed by matings between small queens and small males, indicating stronger selection in bumblebees between partners of similar body masses. Although mating success is influenced by body size, sex ratio, age, and temperature [9,35,37,39], small queens showed an increased tendency to mate with large males at elevated temperatures compared with optimal temperatures. Such temperature-dependent shifts in mate choice may represent a mating strategy favoring the high-quality males, which aligns with observations of mating behavior across species [53,54,55] and in bumblebees [17]. Body size has similarly been shown to influence mating preferences; both queens and males prefer larger mating partners [43,44], a pattern that represents a behavioral adaptation to optimize reproductive success [56]. Furthermore, non-competitive mating experiments conducted at higher temperatures, with equal numbers of small queens and small males, revealed low mutual mate preference between small queens and small males, in contrast to the high mate preference observed between large queens and large males. These results suggest that small males are at a disadvantage in mating trials, particularly at higher temperatures. Overall, our findings show that, under equal sex ratios, selection patterns depend on the body mass of both queens and males across temperatures, and that mating success is influenced by both body mass and temperature, highlighting the strong effects of body mass and temperature on selection and mating success in bumblebees.

Mating success arising from intra- and intersexual selection is a crucial component of the bumblebee life cycles, as mating success determines the survival of future generations. In particular, virgin *B. terrestris* queens typically mate with a single male and subsequently undergo winter hibernation [30]. Therefore, bumblebee queens must store sufficient sperm during mating in their reproductive tracts, along with other male-derived substances [30,57]. Within these selection patterns, a preference for larger mating partners may represent an important strategy for ensuring reproductive success through sexual selection [56]. Although matings between small queens and small males occurred later at both temperatures relative to other selection patterns and exhibited low mutual mate preference at higher temperatures, small males performed longer mating durations than large males, regardless of whether the related mating partners were large or small queens. This suggests that the extended mating durations of small males may facilitate the transfer of more sperm to queens, indicating that small males transferred a higher proportion of their sperm to queens. In addition, within these selection patterns, mating durations at 32 °C were shorter than those at 23 °C, suggesting that elevated temperatures influence mating durations. Nevertheless, large males transferred more sperm to the spermathecae of the queens across both temperatures, and large queens stored more sperm than their smaller counterparts [17]. Since ejaculate quantity is influenced by male body size, large queens may gain reproductive benefits by receiving higher quantities of sperm and seminal fluid substances from large males during mating [45,46,58,59,60,61,62,63]. Similarly, the preference of a queen for large males may enhance sperm viability and overall reproductive success by increasing the transfer of seminal fluid substances, including seminal fluid proteins [41,64,65,66]. Moreover, large queens are more effective at surviving hibernation owing to the possession of sufficient energy reserves [67,68,69,70]. Our previous study demonstrated a positive relationship between body mass and survival during hibernation in mated bumblebee queens, indicating a significantly reduced hibernation survival rate in smaller queens [17]. Thus, our findings suggest that large queens and large males may be positively associated with fecundity benefits, in which the transfer and storage of greater quantities of sperm and seminal fluid are favored.

Therefore, body mass and temperature influence mating success. These results also suggest a potentially concerning scenario in which climate warming affects bumblebee populations. Specifically, climate warming may reduce body size [8,18] as well as mating success [9,17], which, in turn, can reduce mating preferences, mating rates, and hibernation survival [9,17], ultimately contributing to population decline. These findings on bumblebees provide new insights into mating behaviors and reproductive strategies at higher temperatures and have important implications for predicting the fitness of wild bumblebee populations under future climate warming.

## Data Availability

The original contributions presented in this study are included in the article/Supplementary Material. Further inquiries can be directed to the corresponding authors.

## Supplementary Materials

The following are available online at Zenodo: Table S1: Mating behavior and selection outcomes (https://zenodo.org/records/18787841; accessed on 26 February 2026); Figure S1: Mating success rates (%) of LQs, SQs, LMs, and SMs at 23 °C and 32 °C, partitioned by group of mated individuals from the total sample numbers, are shown in Figure 2A (https://zenodo.org/records/19612619; accessed on 17 April 2026); Figure S2: Mating rates (%) under equal numbers of SQs and SMs or LQs and LMs at 32 °C (https://zenodo.org/records/18787811; accessed on 26 February 2026).

## References

[1] Wiens J.J. (2016). Climate-related local extinctions are already widespread among plant and animal species. PLoS Biol..

[2] Martinet B., Dellicour S., Ghisbain G., Przybyla K., Zambra E., Lecocq T., Boustani M., Baghirov R., Michez D., Rasmont P. (2021). Global effects of extreme temperatures on wild bumblebees. Conserv. Biol..

[3] Maebe K., Hart A.F., Marshall L., Vandamme P., Vereecken N.J., Michez D., Smagghe G. (2021). Bumblebee resilience to climate change, through plastic and adaptive responses. Glob. Change Biol..

[4] Gérard M., Amiri A., Cariou B., Baird E. (2022). Short-term exposure to heatwave-like temperatures affects learning and memory in bumblebees. Glob. Change Biol..

[5] Gérard M., Cariou B., Henrion M., Descamps C., Baird E. (2022). Exposure to elevated temperature during development affects bumblebee foraging behavior. Behav. Ecol..

[6] Gérard M., Guiraud M., Cariou B., Henrion M., Baird E. (2023). Elevated developmental temperatures impact the size and allometry of morphological traits of the bumblebee *Bombus terrestris*. J. Exp. Biol..

[7] Perl C.D., Johansen Z.B., Moradinour Z., Guiraud M., Restrepo C.E., Jie V.W., Miettinen A., Baird E. (2022). Heatwave-like events during development are sufficient to impair bumblebee worker responses to sensory stimuli. Front. Ecol. Evol..

[8] Kim H.S., Kim J.M., Qiu W., Yoon H.J., Lee K.Y., Lee K.S., Jin B.R. (2024). Negative relationships between elevated developmental temperatures and morphological traits of different castes of bumblebees (*Bombus terrestris*). J. Asia Pac. Entomol..

[9] Kim H.S., Yoon H.J., Kim B.Y., Lee K.Y., Trewick S.A., Lee K.S., Jin B.R. (2025). The impact of experimental warming on bumblebees: Higher temperatures induce behavioral changes in *Bombus terrestris* queens. Entomol. Gen..

[10] Martinet B., Lecocq T., Smet J., Rasmont P. (2015). A protocol to assess insect resistance to heat waves, applied to bumblebees (*Bombus* Latreille, 1802). PLoS ONE.

[11] Sirois-Delisle C., Kerr J.T. (2018). Climate change-driven range losses among bumblebee species are poised to accelerate. Sci. Rep..

[12] Fourcade Y., Åström A., Öckinger E. (2019). Climate and land-cover change alter bumblebee species richness and community composition in subalpine areas. Biodivers. Conserv..

[13] Soroye P., Newbold T., Kerr J. (2020). Climate change contributes to widespread declines among bumble bees across continents. Science.

[14] White S.A., Dillon M.E. (2023). Climate warming and bumble bee declines: The need to consider sub-lethal heat, carry-over effects, and colony compensation. Front. Physiol..

[15] Gérard M., Michez D., Debat V., Fullgrabe L., Meeus I., Piot N., Sculfort O., Vastrade M., Smagghe G., Vanderplanck M. (2018). Stressful conditions reveal decrease in size, modification of shape but relatively stable asymmetry in bumblebee wings. Sci. Rep..

[16] Guiraud M., Cariou B., Henrion M., Baird E., Gérard M. (2021). Higher developmental temperature increases queen production and decreases worker body size in the bumblebee *Bombus terrestris*. J. Hymenopt. Res..

[17] Park M.S., Woo J.H., Yoon H.J., Kim B.Y., Lee K.Y., Trewick S.A., Lee K.S., Jin B.R. (2025). Body mass and mate choice in bumblebees (*Bombus terrestris*) under climate heating. J. Therm. Biol..

[18] del Castillo R.C., Sanabria-Urbán S., Serrano-Meneses M.A. (2015). Trade-offs in the evolution of bumblebee colony and body size: A comparative analysis. Ecol. Evol..

[19] Siepielski A.M., Morrissey M.B., Carlson S.M., Francis C.D., Kingsolver J.G., Whitney K.D., Kruuk L.E.B. (2019). No evidence that warmer temperatures are associated with selection for smaller body sizes. Proc. R. Soc. B Biol. Sci..

[20] Kingsolver J.G., Huey R.B. (2008). Size, temperature, and fitness: Three rules. Evol. Ecol. Res..

[21] Waller J.T., Svensson E.I. (2017). Body size evolution in an old insect order: No evidence for Cope’s Rule in spite of fitness benefits of large size. Evolution.

[22] Parrett J.M., Knell R.J. (2018). The effect of sexual selection on adaptation and extinction under increasing temperatures. Proc. R. Soc. B Biol. Sci..

[23] Cally J.G., Stuart-Fox D., Holman L. (2019). Meta-analytic evidence that sexual selection improves population fitness. Nat. Commun..

[24] Servedio M.R., Boughman J.W. (2017). The role of sexual selection in local adaptation and speciation. Annu. Rev. Ecol. Evol. Syst..

[25] Gómez-Llano M., Scott E., Svensson E.I. (2021). The importance of pre- and postcopulatory sexual selection promoting adaptation to increasing temperatures. Curr. Zool..

[26] Röseler P.F. (1985). A technique for year-round rearing of *Bombus terrestris* (Apidae, Bombini) colonies in captivity. Apidologie.

[27] Vogt D.F. (1986). Thermoregulation in bumblebee colonies. I. Thermoregulatory versus brood-maintenance behaviors during acute changes in ambient temperature. Physiol. Zool..

[28] Yoon H.J., Kim S.E., Lee S.B., Seol K.Y. (2005). The Effect of antiseptic and sugar solution on colony development of the bumblebees, *Bombus ignitus* and *B. terrestris*. Int. J. Indust. Entomol..

[29] Sepúlveda Y., Nicholls E., Schuett W., Goulson D. (2024). Heatwave-like events affect drone production and brood-care behaviour in bumblebees. PeerJ.

[30] Baer B., Morgan E.D., Schmid-Hempel P. (2001). A nonspecific fatty acid within the bumblebee mating plug prevents females from remating. Proc. Natl. Acad. Sci. USA.

[31] Vanderplanck M., Martinet B., Carvalheiro L.G., Rasmont P., Barraud A., Renaudeau C., Michez D. (2019). Ensuring access to high quality resources reduces the impacts of heat stress on bees. Sci. Rep..

[32] Kenna D., Pawar S., Gill R.J. (2021). Thermal flight performance reveals impact of warming on bumblebee foraging potential. Funct. Ecol..

[33] Kuo Y., Lu Y.H., Lin Y.H., Lin Y.C., Wu Y.L. (2023). Elevated temperature affects energy metabolism and behavior of bumblebees. Insect Biochem. Mol. Biol..

[34] Naumchik M., Youngsteadt E. (2023). Larger pollen loads increase risk of heat stress in foraging bumblebees. Biol. Lett..

[35] Boomsma J.J., Ratnieks F.L.W. (1996). Paternity in eusocial Hymenoptera. Philos. Trans. R. Soc. B-Biol. Sci..

[36] Amin M.R., Than K.K., Kwon Y.J. (2010). Mating status of bumblebees, *Bombus terrestris* (Hymenoptera: Apidae) with notes on ambient temperature, age, and virginity. Appl. Entomol. Zool..

[37] Treanore E., Barie K., Derstine N., Gadebusch K., Orlova M., Porter M., Purnell F., Amsalem E. (2021). Optimizing laboratory rearing of a key pollinator, *Bombus impatiens*. Insects.

[38] Mattle B., Wilson A.B. (2009). Body size preference in the pot-bellied seahorse *Hippocampus abdominalis*: Choosy males and indiscriminate females. Behav. Ecol. Sociobiol..

[39] Amin M.R., Bussière L.F., Goulson D. (2012). Effects of male age and size on mating success in the bumblebee *Bombus terrestris*. J. Insect Behav..

[40] Walzer A., Schausberger P. (2015). Interdependent effects of male and female body size plasticity on mating behaviour of predatory mites. Anim. Behav..

[41] Zhang Y., Zhao C., Ma W., Cui S., Chen H., Ma C., Guo J., Wan F., Zhou Z. (2021). Larger males facilitate population expansion in *Ophraella communa*. J. Anim. Ecol..

[42] Zhao H., Mashilingi S.K., Liu Y., An J. (2021). Factors influencing the reproductive ability of male bees: Current knowledge and further directions. Insects.

[43] Andersson M., Krebs J., Clutton-Brock T. (1994). Sexual selection. Monographs in Behavior and Ecology.

[44] Blanckenhorn W.U. (2005). Behavioral causes and consequences of sexual size dimorphism. Ethology.

[45] Gençer H.V., Firatli Ç. (2005). Reproductive and morphological comparisons of drones reared in queenright and laying worker colonies. J. Apic. Res..

[46] Gençer H.V., Kahya Y. (2011). Are sperm traits of drones (*Apis mellifera* L.) from laying worker colonies noteworthy?. J. Apic. Res..

[47] Garcia-Roa R., Garcia-Gonzalez F., Noble D.W.A., Carazo P. (2020). Temperature as a modulator of sexual selection. Biol. Rev..

[48] Pilakouta N., Baillet A. (2022). Effects of temperature on mating behaviour and mating success: A meta-analysis. J. Anim. Ecol..

[49] Yoon H.J., Lee K.Y., Hwang J.S., Park I.G. (2010). Chilling temperature and humidity to break diapause of the bumblebee queen *Bombus terrestris*. Int. J. Indust. Entomol..

[50] Brodie E.D., Moore A.J., Janzen F.J. (1995). Visualizing and quantifying natural selection. Trends Ecol. Evol..

[51] Conrad T., Stöcker C., Ayasse M. (2017). The effect of temperature on male mating signals and female choice in the red mason bee, *Osmia bicornis* (L.). Ecol. Evol..

[52] Crespi B.J. (1989). Causes of assertive mating in arthropods. Anim. Behav..

[53] Candolin U., Heuschele J. (2008). Is sexual selection beneficial during adaptation to environmental change?. Trends Ecol. Evol..

[54] Martinossi-Allibert I., Rueffler C., Arnqvist G., Berger D. (2019). The efficacy of good genes sexual selection under environmental change. Proc. R. Soc. B Biol. Sci..

[55] Matzke M., Rossi A., Tuni C. (2023). Pre- and post-copulatory sexual selection increase offspring quality but impose survival costs to female field crickets. J. Evol. Biol..

[56] Evans J.P., Garcia-Gonzalez F. (2016). The total opportunity for sexual selection and the integration of pre- and post-mating episodes of sexual selection in a complex world. J. Evol. Biol..

[57] Chapman T., Liddle L.F., Kalb J.M., Wolfner M.F., Partridge L. (1995). Cost of mating in *Drosophila melanogaster* females is mediated by male accessory gland products. Nature.

[58] Ebbert M.A. (1998). The evolution of mating systems in insects and arachnids. Ann. Entomol. Soc. Am..

[59] Harai A.R., Handler A.M., Landolt P.J. (1999). Size-assortative mating, male choice and female choice in the curculionid beetle *Diaprepes abbreviates*. Anim. Behav..

[60] Schlüns H., Schlüns E.A., Van Praagh J., Moritz R.F. (2003). Sperm numbers in drone honeybees (*Apis mellifera*) depend on body size. Apidologie.

[61] Crean A.J., Adler M.I., Bonduriansky R. (2016). Seminal fluid and mate choice: New predictions. Trends Ecol. Evol..

[62] Hopkins B.R., Sepil I., Wigby S. (2017). Seminal fluid. Curr. Biol..

[63] Macartney E.L., Crean A.J., Kakagawa S., Bonduriansky R. (2019). Effects of nutrient limitation on sperm and seminal fluid: A systematic review and meta-analysis. Biol. Rev..

[64] den Boer S.P., Sturup M., Boomsma J.J., Baer B. (2015). The ejaculatory biology of leafcutter ants. J. Insect Physiol..

[65] Kim Y.H., Kim B.Y., Yoon H.J., Choi Y.S., Lee K.S., Jin B.R. (2024). Amwaprin is a sperm-binding protein that inhibits sperm motility and enhances sperm viability in honeybees. Entomol. Gen..

[66] Kim J.M., Kim B.Y., Kim Y.H., Yoon H.J., Choi Y.S., Lee K.Y., Kim D.W., Lee K.S., Jin B.R. (2026). The role of the Niemann-Pick type C2 protein as a sperm-binding protein in honeybees. Insect Biochem. Mol. Biol..

[67] Hahn D.A., Denlinger D.L. (2007). Meeting the energetic demands of insect diapause: Nutrient storage and utilization. J. Insect Physiol..

[68] Hahn D.A., Denlinger D.L. (2011). Energetics of insect diapause. Annu. Rev. Entomol..

[69] Vesterlund S.R., Lilley T.M., van Ooik T., Sorvari J. (2014). The effect of overwintering temperature on the body energy reserves and phenoloxidase activity of bumblebee *Bombus lucorum* queens. Insectes Sociaux.

[70] Keaveny E.C., Dillon M.E. (2022). Phat queens emerge fashionably late: Body size and condition predict timing of spring emergence for queen bumble bees. Insects.

